# Prevalence of Depression and Associated Factors Among Older People in Gezira State, Sudan: A Cross Sectional Study

**DOI:** 10.1002/hsr2.70589

**Published:** 2025-03-20

**Authors:** Aamir Ahmed, Sahar Hamid

**Affiliations:** ^1^ Department of Applied Statistics and Demography Faculty of Economic and Rural Development, University of Gezira Wad Madani Sudan; ^2^ Department of Psychology Faculty of Education, University of Gezira Wad Madani Sudan; ^3^ Department of Applied Psychology Faculty of Education, University of Gezira Wad Madani Sudan

**Keywords:** depression, elderly, prevalence, Sudan

## Abstract

**Background and Aims:**

Sudan's ageing population is growing rapidly, yet research on the psychological health and depression mainly among the elderly is limited. Ageing research is urgently needed to offer critical data for policy formation and program implementation. This study was aimed to address the prevalence and associated factors of depression among older people in Gezira state, Sudan.

**Methods:**

A cross‐sectional study was conducted to collect data from older adults at the household level in Gezira State between January and December 2021. The 15‐item Geriatric Depression Scale‐15 was utilized to facilitate face‐to‐face interviews with a sample of 1068 participants. Both descriptive and inferential statistical methods, including the Chi‐square test and logistic regression analysis, were employed to analyze the data.

**Results:**

A total of 1068 elderly individuals participated in the study, with a depression prevalence rate of 44%. Several factors were found to be significantly associated with depression, including illiteracy (OR = 1.411, 95% CI [1.030–1.933]), being unmarried (OR = 1.500, 95% CI [1.071–2.099]), having diabetes (OR = 1.489, 95% CI [1.102–1.940]), and heart disease (OR = 1.902, 95% CI [1.001–3.614]). Additional factors included disability (OR = 2.360, 95% CI [1.683–3.310]), poor self‐rated health (OR = 1.900, 95% CI [1.426–2.533]), lack of regular contact with relatives (OR = 1.595, 95% CI [1.073–2.371]), and consuming fewer than three meals per day (OR = 2.882, 95% CI [0.942–8.818]).

**Conclusion:**

The findings of this study contribute valuable new data to the field of geriatric health, particularly in the area of psychological well‐being. Building upon previous research, the results of the current study can inform the development of strategies aimed at mitigating depression among the elderly population in Sudan.

## 1 Introduction

Globally, the population is aging quickly. Currently 900 million people aged 60 and over, with this figure expected to double from 12% to 22% by 2050, equating to nearly 2 billion [1, 2, 3]. Similarly, the adult population over the age of 60 across Africa, including Sudan, is predicted to grow from 48 million to 207 million by 2050 [1]. The rate of population aging varies by country due to differences in demographic transition stages, which are linked to economic and social growth. According to population censuses, Sudan's population aged 60 and up grew from 2.5% to 5% in less than 30 years, with a projected 7.5% in 2021 [4, 5]. The expanding elderly population is a result of the demographic transformation that has occurred during the last three decades. Sudan is currently entering stage three of the demographic transition. This stage's characteristics include decreased birth and mortality rates. The Total Fertility Rate in Sudan declined from 5 in 2008 [4] to 4.3 in 2021 [5], and life expectancy at birth increased from 60 years in 2008 to 66 years in 2021 [5], leading to an increase in the number and proportion of older population. Aging is a normal phenomenon that appears with its challenges and is thus a public health concern. The certain reduced of functionalities begin to experience as individual get advances in age [6].

Depression is the primary cause of mental health‐related morbidity and mortality globally [3, 7, 8, 9, 10]. While depression could affect anyone in all age levels, it is more likely to be spread among older population affecting 7% of those aged 60 and above globally [3, 11] and accounting for 5.7% of the individual's years of living with disability (YLD) [3, 12]. In 2015, it was estimated that 800,000 older adults committed suicide annually, and depression is the single most significant contributor to global disability 97.5% [13]. Around the world, approximately 322 million people get affected with depression with the worldwide prevalence of the range 10%–20% depending on cultural situations [14, 15]. The burden of depression among older population in developed countries like America, England and Australia estimated at (9.8%–11.2%) [6, 16], (8.7%) [17], and (8.2%) [18] respectively. Other estimates from the West suggest that up to 16% of its population shows depressive symptoms [19, 20]. In contrast of these figures the high prevalence of depression present in Africa; for instance, in Ethiopia, the burden ranged between 28.5% and 45% [21, 22, 23] 37.5%–44.4% in Egypt [24, 25] 19%–29.3% in Uganda [26, 27] 44.7% in Nigeria [28].

Sudan, like many other developing countries, continues to struggle to provide its people with the most basic psychological and social support services, as well as health services, to help them overcome the vulnerability that may arise as a result of unpredictable social, economic, and political changes. The health status of older persons in Sudan focuses on communicable and environmental diseases rather than psychologically generated diseases, psychosomatic ailments, and those associated to loneliness/not feeling lonely.

To the best of our knowledge, limited research has been conducted on the elderly population in Sudan, particularly in the realm of mental and psychological health. Furthermore, studies at the state level remain scarce. A notable study by S.M. Assil and Z.A. Zeidan (2013) [29] examined the prevalence of depression among the elderly in Khartoum state. The results indicated a depression prevalence rate of 41.1%. Significant factors associated with depression included age, educational level, occupation, daily living challenges, employment status, social issues, and urinary disorders. Additionally, another cross‐sectional descriptive study explored the prevalence of depression among Sudanese patients with type‐2 diabetes mellitus attending a medical insurance clinic in Omdurman [30]. This study enrolled 400 patients diagnosed with type‐2 diabetes, of whom 176 (44%) exhibited symptoms of depression. Among those with depression, 52.3% experienced mild depression, 29.5% suffered from severe depression, and 18.2% had moderate depression.

A review of the aforementioned studies reveals that all were conducted within Khartoum state, with the second study focusing on a specific cohort of individuals with diabetes. The first study addressed a limited number of variables, neglecting other critical factors such as noncommunicable diseases, nutritional status, lifestyle, and others. Given these limitations, there is a clear need for further empirical investigations into the prevalence and determinants of depression, aimed at generating a robust data set that can inform policymakers, researchers, and healthcare providers. Consequently, the present study seeks to estimate the prevalence of depression and identify the factors associated with depression in community‐dwelling older adults in Gezira state, located in central Sudan.

## 2 Methods

### 2.1 Design and Setting of the Study

A cross‐sectional study design was employed to collect data from older people at the household level in Gezira State from January to December 2021. The setting for this study, Gezira State, is in Central of the Sudan. Gezira State is administratively divided into eight localities (Greater Medani, South of Al‐Gezira, East of Al‐Gezira, Al‐Managil, Algorashe, Al‐Kamleen, The Hesahesa, and Umm Al‐Qura), and 43 administrative unit (local administrative unit below locality) and has an area of 23,373 km^2^ and a population of approximately 3.6 million according to population census 2008.

### 2.2 Sample Size and Data Collection

The sample size for the study was estimated using the Cochrane formula: *n* = z*p(1−p)/d2 [31]. S.M. Assil and Z.A. Zeidan's Khartoum study [29] found a 41.8% prevalence of depression in the elderly. However, to maximize the sample size, the prevalence of depression was estimated to be 50%. As a result, a sample size of 600 was determined using *p* = 0.50, a 95% confidence interval, *z* = 1.96, and a 4% margin of error. For cluster sampling, with design effect of 2, the sample size was calculated to be 1200. This analysis involved 1068 individuals, given a response rate of approximately 90%. Cluster sample was used to distribute the study sample among the elderly in Gezira State. The cluster was based on a primary sampling unit (PSU) that is determined by geographic information such as the school or the mosque (a prominent landmark) that directly helps in inference and access to the place of the interview. Within the cluster, the pathway followed by the researcher was determined through the method of Kish. The first question asked by the interviewer was, whether a person aged 60 and over was living in this house and if yes, the elder person was asked to fill the questionnaire. If there was no elderly in that house, the interviewer will move to the next house according to Kish's Table.

The first question asked by the interviewer was whether a person aged 60 or older was living in the household. If yes, the elderly person was asked to complete the questionnaire. If no elderly individual resided in the house, the interviewer would proceed to the next household according to Kish's Table. The interview was conducted by a team of 40 interviewers, all of whom were either university students or graduates. The interviewers received training from the researchers over 2 days of 4‐h sessions. The training covered the survey's purpose, the importance of the interviewer's role, key concepts, how to locate elderly individuals, how to approach the elderly person in their home, and specific guidance on how to conduct the interview—how to begin, carry out, and conclude it.

### 2.3 Study Questionnaire

Face‐to‐face structured interviews were conducted using a structured questionnaire. The questionnaire was initially designed in English and subsequently translated into Arabic. The final structured interview was organized into eight sections: demographics, health status and diseases, lifestyle factors, socioeconomic status, functional status, environmental factors, nutritional status, and depression assessment using the Geriatric Depression Scale (GDS).

### 2.4 Ethical Consideration

This study has been granted ethical approval by the University of Gezira Ethical Review Board. Oral informed consent was obtained from all participants before data collection, ensuring their full understanding of the study's purpose, procedures, and confidentiality safeguards.

### 2.5 Study Variables

#### 2.5.1 Dependents Variable

“Depression status” was adopted as a dependent variable in this study. Depression was measured using GDS [32]. GDS is easy to administer in different settings including the household levels to assess depression. It has been widely evaluated in low‐middle‐income countries such as Ethiopia, India, Europe and other Asian countries [22, 33, 34, 35]. and it is stated 92% and 89% of sensitivity and specificity in the Asian and European adult populations respectively [33, 34]. The overall score for this scale is 15 points and each item has a binary response of “Yes” or “No.” Five of the scale's items are negative questions. So, if participants answer yes, it is coded as “0,” and if they state no, it is coded as “1.” The remaining positive GDS item 15 questions, on the other hand, were classified as “1” if yes and “0” if no. The depression scale is categorized based on the cut‐off scores which are “Normal” (1–5 scores) and “Depressed” (6–15 scores) [23, 36].

#### 2.5.2 Explanatory Variables

Demographic factors as independent variables in this study included age, gender (male, female), family type (extended, nuclear), education and residence (urban and rural). Age was grouped as: (60–79), and (80 and above). Educational status was categorized as illiterate and educated. Educated included those who have passed primary level, intermediate, higher secondary level, bachelors and above.

Lifestyle factors in this study include physical exercise, obesity, smoking habits, and alcohol consumption. Physical exercise was assessed by asking whether the respondent performs regular exercise or never exercises. Smoking habits and alcohol consumption were assessed with the questions, “Do you smoke (yes/no)?” and “Do you consume alcohol (yes/no)?” Obesity status was self‐reported (yes/no). Nutritional status was determined based on meals consumed during the day. Those who consumed ≤ 1 meal per day were considered to have malnutrition.

The presence of diseases was based on self‐reports of being medically diagnosed (by a doctor) with diabetes, hypertension, heart disease, or arthritis. Self‐Rated Health (SRH) was assessed by the question: “How do you rate your general health?” with the responses: “very good,” “good,” “moderate,” “not good,” and “very bad.” For analysis purposes, the responses were collapsed into “Good” (very good and good) and “Poor” (moderate, not good, and very bad). Functional limitations were assessed via self‐reporting.

The Katz Index of activities of daily living (ADL) was used to evaluate disability, including bathing, feeding, dressing, transferring, and toileting [37]. Respondents were considered “disabled” if they reported being unable to perform one or more of these activities independently, without aids.

Social participation was measured by asking whether the individual engaged in any social activities in the past month, such as providing help to family, friends, or neighbors, doing voluntary or charity work, participating in community organizations, or caring for a sick or disabled adult who does not live with them. Those who responded “none of these” were recorded as “0 = no,” and all other choices were recorded as “1 = yes.” For friend and relative contact, the questions were: “Did you interact with your friends?” and “Did you visit your relatives?” Each had six alternatives, classified as either “yes” (daily, once a week, once a month, once a year) or “no” (never). The physical condition of homes can increase the risk of diseases and depression. Four factors were considered: the source of drinking water, sanitation, the number of rooms (one room or more), and durable goods (≤ 2 or more than 2). Drinking water sources were classified into two categories: safe (tap water or boreholes) and unsafe (covered well, uncovered well, tanker, or surface water). Sanitation was categorized as good (siphon or pit latrine) or poor (well bucket).

### 2.6 Statistical Analysis

SPSS version 20 for Windows was used for the statistical analysis. Frequencies and percentages were used to find the frequency distribution of the study variables. Chi‐squared test was used as a preliminary analysis to identify variables for inclusion in the logistic model. Results were reported as frequencies and percentages, with a two‐tailed *p* value of < 0.01 considered statistically significant. To determine the most critical factors associated with depression, multiple logistic regression was performed. The final logistic model included each independent variable found to be significantly associated with depression in the Chi‐squared test at the 1% significance level. The results of the logistic model were reported as an odds ratio (OR), with a 95% confidence interval (95% CI); a two‐tailed *p* value of < 0.05 was considered statistically significant.

## 3 Results

### 3.1 Background Characteristics of the Respondents

Table 1 presents the frequency distributions of the study variables. The sample was distributed between men and women as 59% versus 41%. The majority of the respondents were aged between 60 and 79, and the rest were aged 80 or older. Almost two‐thirds of the elderly were educated. Approximately 65% of the respondents resided in rural areas, and the majority of participants lived in extended family households. Regarding medical diagnoses, 28.7%, 5%, 18.5%, and 26% of older people had diabetes, heart disease, arthritis, and hypertension, respectively. In terms of lifestyle factors, 10.5%, 8%, and 2% of older people were smokers, at risk for obesity, and alcohol addicts, respectively. Additionally, more than 50% of the respondents reported that they exercised irregularly. Table 1 further shows that the prevalence of disability among the elderly was estimated at 23%. In terms of social factors, most of the respondents (86%) maintained contact with relatives, 17% visited their friends, and 40% participated in their community. Regarding the characteristics of the home environment, it is evident that most of the respondents (71%) had improved sanitation, and 47% had unsafe drinking water. Additionally, many older people had more than two durable goods, and three‐quarters of them had more than two rooms in their house. Most of the elderly consumed more than one meal a day. Finally, the prevalence of depression among the elderly was found to be 44%.

**Table 1:** Background characteristics of the elderly.

Variables	Category	Number	Percent
**Dependent variable**			
Depression	Normal	601	56%
	Depressive	467	44%
**Independents variables**			
**Demographic**			
Age	60–79	913	85.5%
	80+	155	14.5%
Gender	Men	632	59%
	Women	436	41%
Education	illiterate	395	37%
	Educated	673	63%
Residence	Urban	380	35.6%
	Rural	688	64.4%
Marital status	Married	765	71.6%
	unmarried	303	28.4%
Family type	Extended	890	83.3%
	Nuclear	178	16.7%
**Health status and diseases**			
Diabetes	Yes	306	28.7%
	No	762	71.3%
Heart disease	Yes	53	5%
	No	1051	95%
Arthritis	Yes	198	18.5%
	No	870	81.5%
Hypertension	Yes	277	26%
	No	791	74%
SRH	Good	705	66%
	Poor	363	34%
**Functional status**			
Disability	Not disable	819	77%
	Disable	249	23%
**Lifestyle factors**			
Use alcohol	No	1046	98%
	Yes	22	2%
Exercise	No	541	50.7%
	Yes	527	49.3%
Obesity	No	982	92%
	Yes	86	8%
Smoking	No	956	89.5%
	Yes	112	10.5%
**Socioeconomic factors**			
Social Participation	Yes	425	40%
	No	643	60%
Relatives contact	Yes	920	86%
	No	148	14%
Friends contact	Yes	181	17%
	No	887	83%
Work status	work	791	74.1%
	not work	277	25.9%
**Home environment**			
Improved sanitation	Yes	308	29%
	No	760	71%
Number durable goods	two or less	283	26.5%
	more than two	785	73.5%
Number of rooms	More than one room	955	89.4%
	one room	113	10.6%
Source of drinking water	Safe drinking water	566	53%
	Un safe drinking water	502	47%
**Nutritional status**			
Meal	More than One	98	98%
	< = 1	19	2%

### 3.2 Distribution of Depression

Table 2 presents the distribution of depression by explanatory variables across the seven sets of factors. The chi‐square test was employed to assess the relationship between depression and the independent variables. The frequency distribution of age, gender, education, and marital status indicated a significant association with depression. In addition, diabetes, heart disease, hypertension, exercise, smoking habits, number of meals per day, and disability were all significantly correlated with depression. Depression was also found to be associated with social activity and interaction with relatives. Home environment variables, such as sanitation, number of rooms, and durable goods, were significantly related to depression.

**Table 2:** Chi‐square analysis of factors associated with depression.

Variables	Depression			Chi‐square values
	Normal	Depressive	Total (*N*)	
**Demographic**				
Age				
60–79	573 (59%)	376 (41%)	913	16.54**
80+	64 (41%)	91 (59%)	155	
Gender				
Men	383 (60.6%)	249 (39.4%)	632	11.78**
Women	218 (50%)	218 (50%)	436	
Education				
Educated	426 (63%)	247 (37%)	673	36.498**
illiterate	175 (44%)	220 (56%)	395	
Residence				
Urban	373 (54%)	315 (46%)	688	3.329
Rural	228 (60%)	152 (40%)	380	
Marital status				
Married	466 (61%)	299 (39%)	765	23.610**
Not married	135 (44.6%)	168 (55.4%)	303	
Family type				
Extended	496 (55.7%)	394 (44.3%)	890	0.6400
Nuclear	105(59%)	73 (41%)	178	
**Health status and diseases**				
Diabetes				
Yes	148 (48.4%)	158 (51.06%)	762	10.89**
No	453 (59.4%)	309 (40.6%)	306	
Arthritis				
Yes	102 (51.5%)	96 (48.5%)	198	2.236
No	499 (57.4%)	371 (42.6%)	870	
Heart Disease				
Yes	19 (36%)	34 (64%)	53	9.45**
No	582 (57%)	433 (43%)	1015	
Hypertension				
Yes	133 (48%)	144 (52%)	277	10.36**
No	468 (59%)	323 (41%)	791	
SRH				
Poor	143 (39.4%)	220 (60.6%)	363	63.67**
Good	458 (65%)	247 (35%)	705	
**Functional status**				
Disability				
Not disable	520 (63.5%)	299 (36.5%)	819	74.391**
Disable	81(32.5%)	168(67.5%)	249	
**Lifestyle factors**				
Use alcohol				
Yes	11 (50%)	11 (50%)	22	0.359
No	590 (56.4%)	456 (43.6%)	1046	
Exercise				
Yes	328 (%)	199 (%)	527	15.05**
No	273 (%)	268 (%)	541	
Obesity				
Yes	46 (53.5%)	40 (46.5%)	86	0.295
No	555 (56.5%)	427 (43.5%)	982	
Smoking				
Yes	69 (61.6%)	43 (38.4%)	112	1.447
No	532 (55.6%)	424 (44.4%)	956	
**Soc‐economic factors**				
Social participation				
Yes	279 (65.6%)	146 (34.4%)	425	25.20**
No	322 (50.1%)	321 (49.9%)	643	
Relatives contact				
Yes	542 (59%)	378 (41%)	920	18.80**
No	59 (40%)	89 (60%)	148	
Friends contact				
Yes	511 (57.6%)	376 (42.4%)	887	3.79
No	90 (49.7%)	91 (50.3%)	181	
Work status				
work	476 (60.2%)	315 (39.8%)	791	18.88**
not work	125 (45.1%)	152 (54.9%)	277	
**Home environment**				
Improved sanitation				
Yes	196 (63.6%)	112 (36.4%)	308	9.53**
No	405 (53.3%)	355 (46.7%)	760	
Number of rooms				
More than one room	522 (58%)	403 (42%)	955	8.56**
one room	49 (43.4%)	64 (56.6%)	113	
Number of durable goods				
two or less	131 (46.3%)	152 (53.7%)	283	15.59**
more than two	470 (60%)	315 (40%)	785	
Source of drinking water				
Safe drinking water	328 (58%)	238 (42%)	566	1.376
Un safe drinking water	273 (54.4%)	229 (45.6%)	502	
**Nutrition status**				
Meal				
More than one	596 (57%)	453 (43%)	1049	7.055**
< = 1	5 (26.3%)	14 (73.7%)	19	

** *p* < 0.01.

### 3.3 Factors Associate With Depression–Logistic Regression

The binary logistic regression analysis presented in Table 3 indicates that elderly individuals who had never attended school, were unmarried, or suffered from diabetes, heart disease, poor self‐rated health, or disability exhibited a significantly higher likelihood of experiencing depression. Additionally, a lack of contact with family members was strongly associated with depression among the elderly. In contrast, no statistically significant associations were observed between depression and variables such as gender, age, hypertension, social participation, smoking, regular exercise, sleep, sanitation, possession of durable goods, number of rooms in the household, and employment status. Older people with no education were 1.411 times more likely to have depression compared to educated elderly individuals (OR = 1.411, 95% CI [1.030–1.933]). Older individuals who had never married were 1.500 times more likely to have depression compared to their married counterparts (OR = 1.500, 95% CI [1.071–2.099]). Older people with diabetes and heart disease were 1.489 and 1.902 times, respectively, more likely to have depression than those without diabetes or heart disease (OR = 1.489, 95% CI [1.030–2.102]; OR = 1.902, 95% CI [1.001–3.614]). Those with poor SRH were 1.90 times more likely to have depression compared to those who perceived their health as good (OR = 1.900, 95% CI [1.426–2.533]). Participants who were unable to perform ADLs independently were 2.36 times more likely to report depression (OR = 2.360, 95% CI [1.683–3.310]). Older individuals who consumed only one meal a day were almost three times more likely to have depression than those who ate more than one meal a day (OR = 2.882, 95% CI [0.942–8.818]). Elderly individuals who did not maintain contact with relatives were 1.595 times more likely to experience depression compared to those with good relationships with their relatives (OR = 1.595, 95% CI [1.073–2.371]).

**Table 3:** Multiple logistic regression analysis of factors associated with depression.

Variable	*B*	SE	OR (95% CI)
Age			
60–79	Ref		1
80+	−0.30	0.210	0.9700 (0.642–1.465)
Gender			
Women	Ref		1
Men	0.244	0.180	0.784 (0.551–1.115)
Education			
Educated	Ref		1
Illiterate	0.344	0.116	1.411 (1.030–1.933)*
Marital status			
Married	Ref		1
Unmarried	0.405	0.172	1.500 (1.071–2.099)*
Heart disease			
No	Ref		1
Yes	0.3398	0.154	1.489 (1.489–1.102)*
Diabetes			
No	Ref		1
Yes	0.643	0.328	1.902 (1.001–3.614)*
Hypertension			
No	Ref		1
Yes	0.198	0.156	1.219 (0.898–1.656)
SHR			
Good	Ref		1
Poor	0.642	0.147	1.900 (1.426–2.533)**
Disability			
Not disable	Ref		1
Disable	0.859	0.173	2.360 (1.683–3.310)**
Smoking			
No	Ref		1
Yes	−0.231	0.232	0.794 (0.503–1.251)
Exercise			
No	Ref		1
Yes	0.206	0.156	1.228 (0.904–1.669)
Sleep			
8 h	Ref		1
Less or more than 8 h	0.011	0.142	1.011 (0.765–1.336)
Social Participation
No	Ref		1
Yes	0.228	0.148	1.256 (0.940–1.679)
Relatives contact			
No	Ref		1
Yes	0.467	0.202	1.595 (1.073–2.371)*
Work status			
Work	Ref		1
Not work	0.116	0.189	1.123 (0.776–1.626)
Improved sanitation			
No	Ref		1
Yes	−0.271	0.160	0.763 (0.558–1.044)
Number of rooms			
More than one room	Ref		1
One room	0.2000	0.230	1.222 (0.779–1.917)
Number of durable goods			
Two or less	Ref		1
More than two	0.191	0.171	1.210 (0.865–1.693)
Meal			
More than one	Ref		1
One	1.059	0.571	2.882 (0.942–8.818)

*Note:* Hosmer and Lemeshow goodness of fit test, Chi‐square = 9.11, df = 8 *p* = 0.333.

Abbreviations: B, unstandardized odds ratio; CI, confidence interval; OR, odds ratio; SE, standard error.

* *p* < 0.05.

** *p* < 0.01.

## 4 Discussions

The present study was an attempt to assess the prevalence of depression and its associated factors among the elderly in the community dwelling in the Gezira State ‐ Sudan. The findings revealed that the prevalence of depression was 44%. This rate is consistent with the rates reported from other community‐based studies conducted in Khartoum ‐ Sudan (41.1%) [29], Nigeria (44.7%) [27], Egypt (44.4%) [38], Ethiopia (41.8%) [22], South Africa (40%) [39], Saudi Arabia (39%) [40], India (42.7%) [41], Pakistan (40.6%) [42] and Iran (43%) [43]. However, this finding was higher 40%, 31.7%, 31.30%, 30.8%, 30.6%, 29.10%, 27%, and 20% than the prior studies conducted in South Africa [11], Europe [44], India [45], China [46], Singapore [47], Netherlands [48], and Mexico [49] respectively. Although the differences are not quite large between them, this may be due to the social, cultural, economic patterns and the standards of living of the countries. Incredibly, the high prevalence reported in the current study compared to the studies of Mexico, India, Singapore, and China could be attributed to differences in the tools used, as the seven‐item version of the Center for Epidemiologic Studies Depression Scale, (ICD‐10), Geriatric Mental State, and Geriatric Depression (ICD‐30) were used to screen depression in Mexico, India, Singapore, and China, respectively. In addition, ages 65–85 were included in the Chinese study, whereas ages 60 and above were used as a cutoff point in our study. The discrepancy in prevalence compared to Europe study may be due to social‐cultural and economic differences. The substantial discrepancy between our study and the South African study can be attributed to the differences in participants, ages as well as socio‐cultural factors. The South African study comprised participants aged 50 and over.

On the other hand, the findings of current study was lower than those of other studies done in, Urban India [50], Malaysia [51], Vietnam [52], Portugal [53] and Japan [54], which reported 80%, 75.3%, 66.9%, 61.4% and 57.2% respectively. These disparities might be ascribed to different reasons. In Portugal and Japan, it might be due to the difference in the study population compared to our target population. The elderly living in institutions may be more depressed as a result of environmental changes after a long period of living in private homes. In Vietnam, the discrepancy could be attributed to tool variation, as they utilized the Zung self‐rating depression scale to screen for depression. In Cuttack India the variation may be due to the tool used for depression assessment, where GSD–30 were applied in. For Malaysia, the disparity could be due to the low socioeconomic status of the specific group that was included in the Malaysian study. The Malaysian study was conducted among those receiving aid from the Penang State government. Additionally, urban environments, offer better access to mental health care and services, besides the better access to resources, and the exciting lifestyle factors which can mitigate depression. On the other hand, in rural areas people face challenges of limited health care and services.

Several factors were found to be related to depression in the current investigation. Elderly individuals who had never attended school, were unmarried, had diabetes or heart disease, were disabled, or reported SRH were significantly associated with depression. Furthermore, older adults who do not maintain contact with family and consume fewer than one meal per day were also found to be related to depression. It's obvious that education is almost associated with cognitive and mental stimulating, better access to healthcare, mental health resources, more awareness of the ability to utilize services that address mental health issues including depression. Compatible with other studies [28, 55, 56, 57, 58] we found a higher burden of depression among the illiterate elderly. Additionally, the burden of depression was found to be higher among unmarried participants. In figures, unmarried participants were 1.50 times more likely to record depression compared to those who were married at the time of the survey. These findings are consistent with similar studies conducted in India [33], Ethiopia [21], and Uganda [26]. Another previous study found that never‐married, divorced, and widowed elders were more susceptible to depression than married ones [11, 41, 59, 60, 61, 62], which shows that married elderly people are likely to be much happier, have higher levels of emotional bonding, greater subjective well‐being, and lower depression compared to those who are unmarried, divorced, or separated.

The current study also found that older people with chronic condition such as diabetes and heart disease were respectively 1.489 and 1.902 times more likely to have depression compared with their counterparts. Heart disease like other physical diseases affected negatively in many aspects in elderly life including attitudes and mood due to its symptoms like the difficulty of breathing, swelling in the feet and ankles, abdomen and feeling of being ding. All those symptoms affected negatively in lifestyle habits which revealed depression. This finding is consistent with the previous results in India [63] that demonstrated elders diagnosed with diabetes were 1.15 times more likely to develop depression compared to non‐diabetics. It is also in line with studies in China and Sri Lanka [59, 64] which present that the elderly who had known chronic diseases were at higher odds of developing depression compared to their counterparts. Historically, higher blood glucose “hyperglycemia” is associated with mood manner negatively in the elderly and may cause fatigue due to dehydration with high glucose level leading to excess glucose in urine which may cause electrolytes and loss of water, and it can affect and lead to aggravated mood swing, cognitive disturbances, and decline in general physical health. Moreover, evidence from WHO, indicated that the increase in the prevalence of chronic illness corresponded with the spread leading of emotional problems and depression.

Limitations in daily activities and physical functions dispose older adults to lose their independence, leading to depressive symptoms and grief. These conditions could lead to psychosocial and financial difficulties. Substantial evidence suggested that ADL disability were at a higher risk of depressive symptoms [65]. Chauhan P. and et al. [33] found a strong relation between the prevalence of depression and physical dependence for daily activities. In another study, depression in the elderly was found to be strongly associated with lower score on ADLs scale in three different Asian countries [66]. Egyptian studies also revealed that there was an inverse relationship between ADL and depression [24, 67]. Our study supported the previous works since we found that those who faced difficulties in doing the ADLs were 2.360 times more likely to have depression. The elderly have general deterioration in general physical functions one of which the locomotors system which appears in bones trending to shrink in size and density, weakening them and making them more susceptible to fracture, muscles generally lose strength, endurance, and flexibility, which can affect condition, stability, balance and their daily activities. Furthermore, arthritis with highest pain levels is the more likely to be depressive due to experiencing considerable pain, unrelenting fatigue, movement problems, less activities, and low mood. Activity theory which is one of the psychological theories of aging proposes that the more active and more engaged a person in old age, is the happier he or she will be.

SRH is considered as a predictor of survival and is commonly used as a main variable in studies of the elderly [68]. Bond et al. (2006), examined the prognostic capacity of this measure and reported that SRH was associated with a higher risk of death, and functional and cognitive impairment [69]. This study showed that, those rating their health as poor were approximately two times more likely to have depression compared to elders perceived themselves as good. Similar findings were found in a study carried out in South Africa, where depression spread among older people with poor subjective health [39]. Also, previous studies revealed that, those rating their health as poor to fair were at risk of depression compared to those rating their health as good to excellent [70, 71, 72]. In addition, two studies suggest that SRH may be an important predictor of poor depression outcome [71, 73]. The ABC model of depression explained that obviously there were activity events for negative situation, such as the deterioration that happen due to age, occur and supported by beliefs, which is mainly irrational believes, explain the situation in a pessimistic and negative thought that lead to consequences in feelings and behavior in response to adversity due to bad believes, so depressed people developed low self‐health rating.

Absence of family support is highly correlated with geriatric depression, with a possible bidirectional interaction [74, 75]. The absence of close family members or a lack of social connections are both regarded as key predictors of depression in the elderly [74, 75, 76].

Consistent with a prior study [55], our investigation found that those who did not maintain regular contact with relatives were more likely to experience geriatric depression. Additionally, good family contact and relationships can help maintain a sense of overall mental well‐being by providing safety, security, love, and belongingness, thus reducing the risk of mental health conditions. On the other hand, poor family contact, disrupted relationships, and a loss of community connections with family members reduce the quality of life and contribute to depression. Another study found that elderly individuals without family support were 1.48 times more likely to develop depression than others [63]. Previous studies in Sri Lanka and India indicated that insufficient social support was one of the contributing variables to depression among older populations [59, 77], which is consistent with our findings. Family support strengthens the elderly by creating feeling reassurance of being cared, safe, beloved and not lonely and they may provide resources that can help them to deal with stress and develop high self‐esteem, which leads to less stress and high well‐being.

## 5 Limitation

Certain GDS components could not be confirmed with the community's cultures and beliefs around fate and destiny, which could lead to bias in response. Additionally, their beliefs may have influenced their responses regarding alcohol consumption and cigarette smoking, leading to social desirability bias, even though the interview was conducted in a separate room. Furthermore, data on obesity status and physical exercise habits in this study were obtained through self‐reports, which are susceptible to inaccuracies. These limitations should be carefully considered in the design of future research, particularly within the Sudanese context at both local and national levels.

## 6 Conclusion

This study found a higher prevalence of depression among the elderly compared to earlier studies in Sudan. Depression was significantly associated with elderly individuals who had never attended school, were unmarried, had diabetes, heart disease, disabilities, and poor SRH. Furthermore, older adults who had limited contact with family and consumed fewer than one meal per day were also found to be more likely to experience depression. Given that depression was more prevalent among older adults with diabetes, heart disease, and disabilities, efforts should focus on addressing these conditions and enabling older adults with disabilities to actively participate in society. Establishing a geriatric care center and ensuring adequate social support could be crucial in alleviating the suffering of the elderly due to depression resulting from a lack of social support.

## Author Contributions

**Aamir Ahmed:** methodology, software, data curation, formal analysis, writing – original draft, conceptualization, project administration, resources, supervision. **Sahar Hamid:** investigation, validation, visualization, writing – review and editing.

## Conflicts of Interest

The authors declare no conflicts of interest.

## Transparency Statement

The lead author Sahar Hamid affirms that this manuscript is an honest, accurate, and transparent account of the study being reported; that no important aspects of the study have been omitted; and that any discrepancies from the study as planned (and, if relevant, registered) have been explained.

## Data Availability

The data that support the findings of this study are available from the corresponding author upon reasonable request.
